# Complex implementation factors demonstrated when evaluating cost-effectiveness and monitoring racial disparities associated with [^18^F]DCFPyL PET/CT in prostate cancer men

**DOI:** 10.1038/s41598-023-35567-w

**Published:** 2023-05-23

**Authors:** Kritika Subramanian, Juana Martinez, Sandra Huicochea Castellanos, Jana Ivanidze, Himanshu Nagar, Sean Nicholson, Trisha Youn, Jones T. Nauseef, Scott Tagawa, Joseph R. Osborne

**Affiliations:** 1 Division of Molecular Imaging and Therapeutics, Department of Radiology, Weill Cornell Medicine, New York, NY USA; 2 Department of Radiation Oncology, Weill Cornell Medicine, New York, NY USA; 3 Department of Policy Analysis and Management, Sloan, Cornell Institute for Public Affairs, New York, NY USA; 4 Department of Medical Oncology, Weill Cornell Medicine, New York, NY USA

**Keywords:** Molecular medicine, Urological cancer

## Abstract

Prostate cancer (PC) staging with conventional imaging often includes multiparametric magnetic resonance (MR) of the prostate, computed tomography (CT) of the chest, abdomen, and pelvis, and whole-body bone scintigraphy. The recent development of highly sensitive and specific prostate specific membrane antigen (PSMA) positron emission tomography (PET) has suggested that prior imaging techniques may be insufficiently sensitive or specific, particularly when evaluating small pathologic lesions. As PSMA PET/CT is considered to be superior for multiple clinical indications, it is being deployed as the new multidisciplinary standard-of-care. Given this, we performed a cost-effectiveness analysis of [^18^F]DCFPyL PSMA PET/CT imaging in the evaluation of PC relative to conventional imaging and anti-3-[^18^F]FACBC (^18^F-Fluciclovine) PET/CT. We also conducted a single institution review of PSMA PET/CT scans performed primarily for research indications from January 2018 to October 2021. Our snapshot of this period of time in our catchment demonstrated that PSMA PET/CT imaging was disproportionately accessed by men of European ancestry (EA) and those residing in zip codes associated with a higher median household income. The cost-effectiveness analysis demonstrated that [^18^F]DCFPyL PET/CT should be considered as an alternative to anti-3-[^18^F]FACBC PET/CT and standard of care imaging for prostate cancer staging. [^18^F]DCFPyL PET/CT is a new imaging modality to evaluate PC patients with higher sensitivity and specificity in detecting disease than other prostate specific imaging studies. Despite this, access may be inequitable. This discrepancy will need to be addressed proactively as the distribution network of the radiotracer includes both academic and non-academic sites nationwide.

## Introduction

Prostate cancer (PC) is the most prevalent cancer among men in the United States, affecting ~ 6 out of every 10 men above the age of 65^1,2^. The incidence of aggressive (versus indolent) and metastatic disease at diagnosis is also rising^3^. The Centers for Medicare and Medicaid services (CMS) currently covers prostate specific antigen (PSA) testing and digital rectal examination (DRE) once a year for all men with Medicare above the age of 50 years^4^ for PC screening. Medicare and Medicaid are federal government run health insurance programs in the United States which primarily provide coverage to senior citizens above the age of 65 and individuals with limited income respectively. Consequently, men above the age of 50 have more access to PSA screening relative to their non-Medicare holding younger counterparts. Unfortunately, the steady increase in incidence of PC diagnoses with serial prostate specific antigen (PSA) testing has also had the effect of augmenting this age-related discrepancy. As an example, those who were uninsured prior to Medicare coverage have demonstrable system-derived cost and access barriers at an earlier stage of the cancer. In fact, this “Medicare effect” resulted in a spike of cancer diagnoses, including prostate cancer, around the age of 65 years in a review of data from 2004 to 2016 extracted from the Surveillance, Epidemiology, and End Results (SEER) database^5^.

Pursuit of medical care may be delayed due to a variety of non-medical reasons, including cost concerns and mistrust of the health care system. A study that surveyed 378 men at a single institution with PC, among whom 38% were African American (AA), found that the odds of decisional regret were greater for AA men than for their European ancestry (EA) counterparts. Furthermore, this was attributed to a greater level of medical mistrust and to masculinity scores^6^. A retrospective cohort study conducted in the US Veterans Health Administration Health Care system found that AA patients with Medicare-only had higher PSA levels at diagnosis^7^, a more aggressive stage of disease^8,9^, and a 10-year cumulative incidence of disease progression^10^. As AA men were more likely to have intermediate-risk disease at diagnosis, they were less likely to be recommended conservative management^11^. Overlap between patient demographics and socioeconomic status may explain why population-level differences in survival are seen in geographic stratification of the SEER data set^12,13^. Conversely, it is known that these disparities in outcome are erased when AA men are treated in a clinical trial environment^14,15^. It is imperative, then, to identify drivers of disparity, such as cost and care access, to address disparities rooted in inequitable treatment.

Imaging is critical in the staging of patients at initial diagnosis as well as biochemical recurrence because the quantification of disease burden, distribution, and subsequent progression determines treatment management. Imaging, along with other visual and informational aids, enhances the understanding of disease involvement and can guide patient decision making^16^. Staging for PC through imaging has conventionally involved multiparametric MR of the prostate for localized disease, and computed tomography (CT) chest, abdomen, and pelvis, with whole body bone scintigraphy for evaluation of extra-pelvic disease^17^. As the sensitivity for early metastatic disease detection is low on conventional imaging, positron emission tomography/computed tomography (PET/CT) imaging using PC-approved tracers such as anti-3-[^18^F]FACBC (^18^F-Fluciclovine) are conjunctionally performed. Combined, the multitude of imaging studies, consequential diagnoses, and overall associated costs may adversely impact the decision to receive medical care.

SEER-Medicare data for PC patients from 2004 to 2007 showed an over-utilization of bone scans in patients with low- and intermediate-risk PC, resulting in excessive and unnecessary Medicare costs. Meanwhile, there is an underutilization of bone scans in high-risk patients with likely metastatic disease^18^. Multiple studies have demonstrated the utility of PET/CT imaging using radioligand-tagged prostate specific membrane antigen (PSMA) at initial staging and biochemical recurrence^19,20^. Internationally PSMA PET/CT has been demonstrated to be a costly imaging modality, but cost-effective when compared to conventional imaging for initial staging^21,22^. The most commonly studied PSMA PET radiopharmaceutical is [^68^Ga]Ga-PSMA-11. PSMA PET/CT independently can save time and act as a decision-making aid for the patient, potentially improving delivery of appropriate care for men with PC. Unfortunately, access to [^68^Ga]Ga-PSMA-11 is limited^23^, such that four times more non-Black patients were likely to obtain a [^68^Ga]Ga-PSMA-11 scan relative to their Black counterparts, likely due, in part, to insurance coverage and out-of-pocket costs in the United States. [^18^F]DCFPyL PET/CT may be superior to [^68^Ga]Ga-PSMA-11 in identifying involved nodes and distant metastases^24^, making it a preferred PSMA radiopharmaceutical agent for prostate cancer PET Imaging. In January 2022, the Food & Drug Administration (FDA) approved the PSMA PET radiopharmaceutical piflufolastat F-18 (also known as [^18^F]DCFPyL or PyL) for commercial use^25^. CMS granted temporary pass-through payment status the same month, followed by approval for contractor determined coverage in May 2022^26,27^. CMS coverage of [^18^F]DCFPyL PET/CT can help address the inequitable access to PC imaging.

In this paper, we review the cost benefits of using PSMA PET, specifically [^18^F]DCFPyL because of its FDA and CMS approval status at the time of this analysis, in a cost-effectiveness analysis, while also analyzing the patient demographics of PC patients who underwent a PSMA PET/CT scan at our institution. In congruence with studies demonstrated in Australia and Europe^21,22^, we hypothesize that PSMA PET/CT has the potential to be more cost-effective than anti-3-[^18^F]FACBC PET and conventional imaging in the United States.

## Methods

### Patient demographics

Patients with PC who underwent a PSMA PET/CT scan (either [^68^Ga]Ga-PSMA-11 or [^18^F]DCFPyL) between January 1, 2018 and December 15, 2021 were extracted from the imaging system. This study was approved by the Weill Cornell Medicine Institutional Review Board (IRB # 1706018301). Informed consent as designated by the IRB was obtained for all study subjects. All research was performed in accordance with the institutional guidelines. Patient age, self-reported ethnicity, and zip code were collected. Zip codes were used as an alternative measure for socioeconomic status by extracting publicly available 2020 census data and stratifying them into six range groups based on median household income for each zip code: < $25k, $25–$50k, $50–$75k, $75–$100k, $100–$200k, and > $200k. Patients were additionally stratified into three groups based on whether they received access to the PSMA PET/CT scan through a research grant, paid out of pocket, or used insurance.

### Evaluating nationwide access to [^18^F]DCFPyL PET/CT

[^18^F]DCFPyL was the only commercially available, FDA-approved PSMA PET/CT radiotracer available in the United States that was also approved for coverage by the CMS and private insurance at the time this analysis was conducted. For this reason [68Ga]Ga-PSMA-11 was not considered in this analysis. After extracting the latitude and longitude dimensions for each zipcode and geographic identifier (GEOID) using the online database Simplemaps^28,29^, geospatial maps of locations where [^18^F]DCFPyL PET/CT were performed as of December 2021 (provided by Lantheus, Billerica, MA), the national distribution of ethnicity from the 2020 census, and adjusted gross income from 2018 IRS tax return data for each zip code were created using the open source Kepler.gl software^30^. For the purpose of geomapping income levels, adjusted gross income data for each zip code from the 2018 IRS tax return data were extracted as counts for each of the six range groups: < $25k, $25–$50k, $50–$75k, $75–$100k, $100–$200k, and > $200k. The counts for each income range group were converted to percentage, where a percentage greater than 20% represented a majority for the zip code.

### Cost-effectiveness analysis

**Figure 1:**
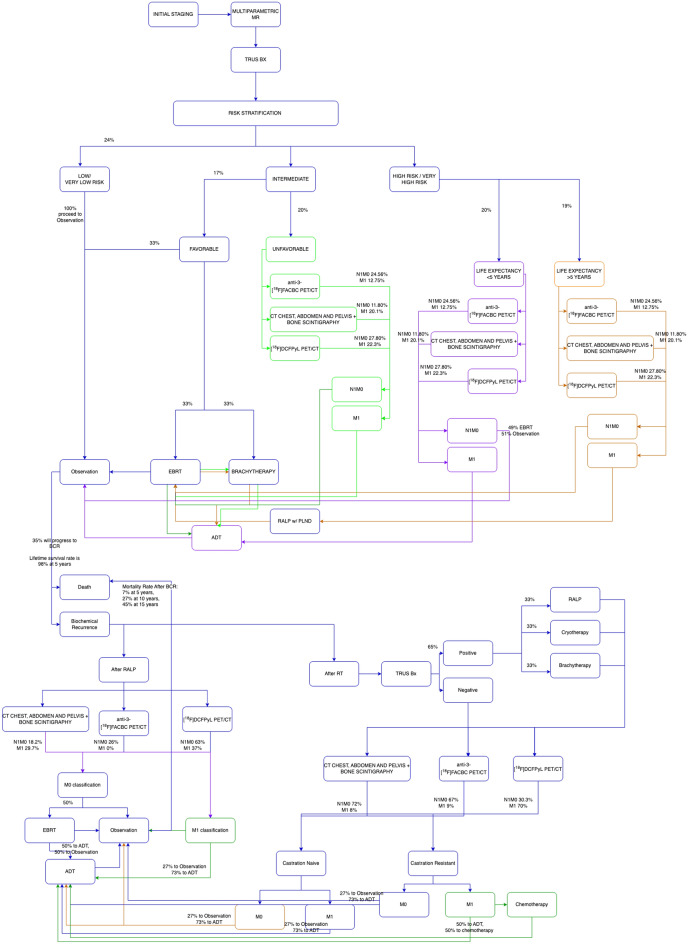
Health states and therapeutic options are defined in this flowchart which was subsequently utilized for the simulation model.

A comprehensive flowchart was first formatted based on 2021 NCCN risk stratification guidelines^31,32^ that depicted the possible course of disease after the diagnosis of PC (Fig. 1). A decision-analytical simulation was developed on GNU OCTAVE (version 6.4.0)^33^ based on Markov chain modeling where health transitions from one state to another were defined through set proportions, time intervals, or a combination of both. Probabilistic sensitivity analysis was performed on the simulation to ensure a model convergence with a 95% confidence interval that does not include zero^34^. A total of 2000 iterations was performed for each simulation run using a sample size of 1000 patients, with a maximum age of 90 years (rounded up from 88 years based on a 95% confidence interval from the 2019 actuarial life table provided by the Social Security Administration)^35^. A pay threshold of $50,000 per quality of adjusted life years (QALY) gained was selected for this analysis^36^. Likelihood ratios of changing from one health state to another were extracted from published literature^1,2,37–46^. These estimated proportions of non-imaging related health states were assumed to be the same for each modality regardless of the risk stratification due to a lack of available literature suggesting otherwise. N1M0 detection by anti-3-[^18^F]FACBC PET/CT, conventional imaging, and PSMA PET/CT were 24.56%, 11.80%, and 27.80%, respectively, whereas M1 detection rates were 12.75%, 20.1%, and 22.3%, respectively at initial staging. These percentages changed for patients at biochemical recurrence depending on whether they had a history of robot-associated laparoscopic prostatectomy (RALP) or radiation therapy (RT) (Fig. 1).

In this healthcare-perspective model, conventional imaging with CT of the chest, abdomen, and pelvis along with nuclear bone scintigraphy was compared with anti-3-[^18^F]FACBC PET/CT and [^18^F]DCFPyL PET/CT after initial imaging with multiparametric MR, as demonstrated in Fig. 1. Imaging modalities were used for staging at initial diagnosis and at biochemical recurrence. Per the National Comprehensive Cancer Network (NCCN) guidelines, there were no recommendations for the frequency of imaging studies, and the decision was left to the discretion of the oncologist as adjunct to blood PSA level measurements. Imaging was therefore evaluated only at initial staging and at biochemical recurrence in this simulation model. Transitions from one health state to another, as well as costs and associated health-related quality-of-life (HRQoL), were based on published literature (Table 1). Costs were extracted from the CMS Physician Fee Schedule^47^ tool and incorporated into the simulation. Trogdon et al.^48^ extrapolated Medicare-associated total costs for PC patients within 3 years of diagnosis using the SEER-Medicare study of 49,692 individuals above the age of 70. These extracted total costs were incorporated into our simulation. Multiple studies have attempted to review quality of life based on the functional assessment survey scores (EORTC QLQ-C30) at each health state after the diagnosis of PC. These scores were extracted from the reported global health function portion of the QLQ-C30 survey and incorporated into the simulation whereby the imaging study types were directly correlated with HRQoL (on a scale of 0–100). For the health states that were being evaluated in this study, common United States based surveys such as the Functional Assessment of Cancer Therapy-Prostate (FACT-P) were not consistently available for incorporation. However, the QLQ-C30 scoring system has been validated for intra-country and inter-country analysis in Europe and North America, including the United States^49^.

**Table 1:** Medical and imaging associated costs (in US dollars), associated distribution type for parameterization, and HRQoLs associated with relevant health states used in the simulation.

	Distribution	Costs	Distribution	HRQoL score	95% CI or std dev	Distribution	Time in state	Std error
Initial staging	$\gamma$	1664	–			$\beta$	16 years^61^	8.6 years
Multiparametric MR	$\gamma$	492.69				–		
Trus Bx	$\gamma$	305.66	$\beta$	80.7^62^	[64.6, 96.8]			
Anti-3-[^18^F]FACBC PET/CT	$\gamma$	2059.47	–					
CT chest, abdomen, and pelvis with bone scintigraphy	$\gamma$	1130.05						
[^18^F]DCFPyL PET/CT	$\gamma$	1617.15						
Observation	$\gamma$	1066	$\beta$	97.44^63^	[94.83, 100]	$\beta$; piecewise distribution (survival analysis)	35% to BCR in 10 years^43^; survival distribution: 98% at 5 years^2^	–
EBRT	$\gamma$	15,000	$\beta$	92.31^63^	[87.44, 97.78]	$\beta$	5 weeks^64^	–
Brachytherapy	$\gamma$	14,200	$\beta$	92.31^63^	[87.44, 97.78]	–		
ADT	$\gamma$	2993	$\beta$	92.31^63^	[87.44, 97.78]	$\beta$	3 years^65^	–
RALP w/ PLND	$\gamma$	10,600	$\beta$	95.51^63^	[87.65, 98.72]	–		
Biochemical recurrence (BCR)	–		$\beta$	78.79^66^	[54.82, 100]			
Observation (after BCR)	$\gamma$	1066	$\beta$	71.21^66^	[53.59, 88.83]	Piecewise exponential (survival analysis)	Survival distribution: 93% at 5 years, 73% at 10 years,55% at 15 years^43^	–
Cryotherapy	$\gamma$	13,500	$\beta$	80^67^	–	–		
Chemotherapy	$\gamma$	122,323	$\beta$	77.8^68^	20.0	$\beta$	6 months^69^	–

Costs were extracted from the 2021 CMS Physician Fee Schedule tool^47^ and assumed a 10% variability/standard error using a $\gamma$ distribution. Health Related Quality of life (HRQoL) scores were derived from published literature (translated to 0–100 scale if not done already) and assumed to follow a $\beta$ distribution. Time in each state were also assumed to follow a $\beta$-distribution.

The QLQ-C30 scores are multiplied with a length of time in which patients are likely to remain in that health state to formulate a health state value. It is important to note that the global health function score of the QLQ-C30 is limited in that it is not cancer-specific. For prostate cancer, we noted that preferential symptoms evaluated were not always consistent among the studies which used QLQ-C30 and therefore believed that utilizing the global health function score would be the best option for this analysis. As a result, there was no need to map the cancer-specific criteria from the QLQ-C30 questionnaire to the European Quality of Life with Five Dimensions (EuroQoL 5D) instrument which provides a single, cancer-specific health score^50^. A discount of 3% was applied to the costs and the QALYs as recommended for United States-based cost-effectiveness analyses^51,52^.

The average age of PC diagnosis was 66 years^1^, and was therefore the average starting age of patients in the simulation at initial staging after beginning annual PSA checks at age 50^1^ and continuing in the simulation until death. One month was assumed to be the approximate time interval required for imaging studies to receive pre-authorization from insurance and scheduled for an appointment^53^. Proportions that did not sum to 100% when changing from one state to another were scaled to 100% while maintaining the same relative ratio. Similarly, the time associated with each medical treatment was as follows: 10 years in the observation state until biochemical recurrence, 6 weeks for the treatment duration of external beam radiation therapy (EBRT), 1 day intervention for brachytherapy, cryotherapy, and robot-associated laparoscopic prostatectomy with pelvic lymph node dissection (RALP w/PLND), and 3 years duration on androgen-deprivation therapy (ADT).

A separate economic health model was performed for each imaging modality. Each model was simulated using 1000 patients and 2000 randomized iterations. Simulation output produced an overall interpretation of medical costs, imaging costs, and QALYS averaged per person out of the 1000 included in the simulation. Incremental cost-effectiveness ratios (ICER) were calculated for each pair of imaging modalities by subtracting the difference of the average total costs and dividing it by the difference in mean HRQoLs. Greater effectiveness and lower cost defined dominance of an imaging modality in the analysis.

### Analysis

#### Single-institution demographics

564 PSMA PET/CTs were conducted at this single institution either with [^18^F]DCFPyL or [68Ga]Ga-PSMA-11. 119 (21%) were eligible for research-sponsored studies, whereas 258 (46%) paid out-of-pocket for the study and 186 (33%) were able to obtain the study through insurance. Table 2 compares the age, self-reported ethnicity, and median household income based on zip code for each cohort, which showed that most patients who had a PSMA PET/CT scan at this single institution were of European Ancestry, irrespective of route of payment. There was no difference in age among the three cohorts. More than 55% of men in each PSMA PET/CT cohort resided in a zip code where the median household income was greater than $100,000.

**Table 2:** Demographics of prostate cancer patients who underwent PSMA PET/CT Scans at this single institution in NYC.

Ethnicity/race	Research	Out-of-pocket	Insurance
Cohort size (n)	119	259	186
Non Hispanic/Latino			
AA	9%	3%	4%
EA	59%	74%	44%
Asian	3%	1%	2%
Other	4%	2%	1%
Declined/unknown	1%	0%	2%
Hispanic/Latino			
AA	0%	2%	0%
EA	4%	1%	2%
Asian	0%	0%	1%
Other	0%	0%	1%
Declined/unknown	0%	0%	2%
Declined/unknown			
AA	0%	0%	2%
EA	4%	5%	13%
Asian	0%	0%	1%
Other	0%	3%	1%
Declined/unknown	16%	8%	25%
Median household income (based on zip code) ($)			
< $25,000	0%	1%	0%
$25,000–$50,000	8%	6%	8%
$50,000–$75,000	21%	17%	19%
$75,000–$100,000	8%	6%	8%
$100,000–$200,000	58%	66%	61%
> $200,000	5%	3%	4%
Age (years)			
20–30	0%	0%	0%
30–40	0%	0%	0%
40–50	1%	2%	1%
50–60	5%	7%	9%
60–70	29%	33%	29%
70–80	40%	40%	45%
80–90	18%	17%	15%
90–100	7%	0%	1%

#### Current access to [^18^F]DCFPyL PET/CT imaging

**Figure 2:**
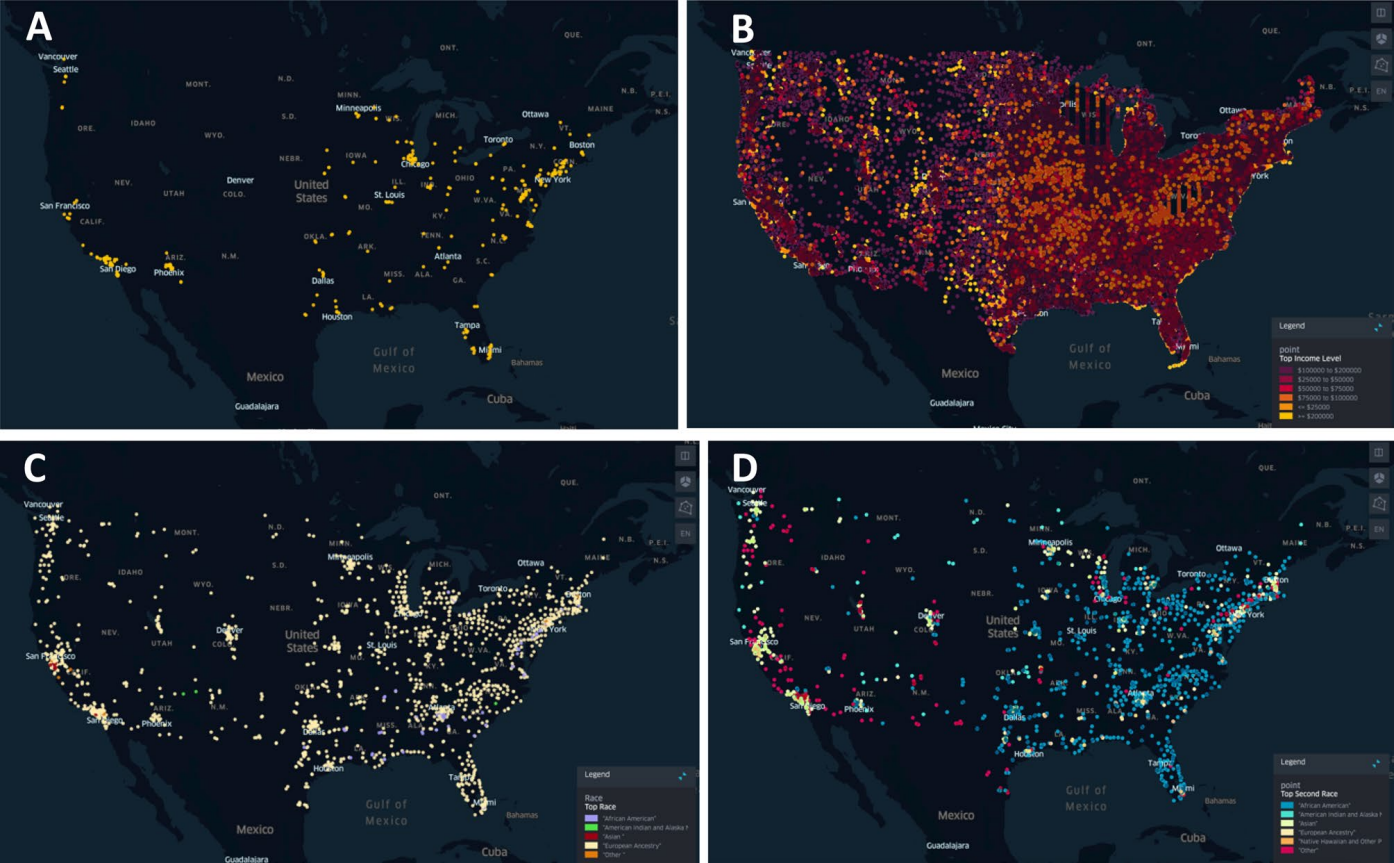
Institutions which are currently offer PSMA scans are predominantly located in the Northeast, Midwest, and the west coast (**A**). These institutions overlapped with zip codes where the residents belonged to a wealthier demographic population (AGI > $100,000) based on data extracted from the 2018 IRS tax returns (**B**) and had an EA majority based on the 2020 Census Data (**C**). When evaluating the second most common ethnic group in these locations (**D**), the east coast had predominantly AA individuals while the West coast was mostly Other and Asians. Geospatial maps created using open source software kepler.gl.

Within a year of entering the United States market, [^18^F]DCFPyL PET/CT was predominantly commercially available in the Northeast, with additional availability in centers in southern California and the Midwest (Fig. 2). Geospatial predominance mapping of race and income within the United States demonstrated [^18^F]DCFPyL offering centers were distributed in areas where most residents were of EA and residing in a neighborhood where the largest proportion of residents (> 20%) had an adjusted gross income greater than $100,000. AAs were the second most common ethnic group in the cities on the east coast where [^18^F]DCFPyL is available, in contrast to the west coast where individuals of “other” ethnicity were the second most abundant.

#### Incremental costs and outcomes

The simulation was run for each imaging modality for a sample size of 1000 individuals using the staging proportions^54–60^ and extracted costs^47^ as outlined in the Methods for a total of 2000 randomized iterations (Supplemental Fig. 1). The findings are described in Table 3. The greatest total cost (medical and imaging combined) was associated with [^18^F]DCFPyL PET/CT imaging at $25,201.73 per person, followed by anti-3-[^18^F]FACBC PET/CT imaging at $18,000.12 per person, and conventional imaging (i.e. CT scan of the chest, abdomen, and pelvis with bone scintigraphy) at $17,176.83 per person. When the cost versus QALY was graphed on a scatterplot, there was high precision for each simulation cohort. [^18^F]DCFPyL PET/CT was the most expensive and associated with higher lifetime composite QALY than anti-3-[^18^F]FACBC PET/CT and conventional imaging. ICER could not be evaluated between anti-3-[^18^F]FACBC PET/CT and conventional imaging as there was no difference in composite lifetime QALY (denominator = 0). The ICER comparing [^18^F]DCFPyL PET/CT with anti-3-[^18^F]FACBC PET/CT and conventional imaging exceeded the willingness-to-pay threshold of $50,000/QALY gained, despite a higher composite lifetime QALY score.

**Table 3:** Mean total costs and HRQoL for 2000 iterations of the simulation at n = 1000 is listed above in the first table.

	Anti-3-[^18^F]FACBC PET/CT	CT C/A/P + bone scan	[^18^F]DCFPyL PET/CT
Total cost ($, u ± sd)	18,000.12 ± 579.32	17,176.83 ± 544.06	25,201.73 ± 1031.74
Composite lifetime QALY (u ± sd)	7.71 ± 0.13	7.71 ± 0.13	7.83 ± 0.13
Total gained years (u ± sd)	19.44 ± 0.26	19.50 ± 0.26	19.55 ± 0.27

Comparison modalities		ICER	
Anti-3-[^18^F]FACBC PET/CT	CT C/A/P + bone scan	–	
[^18^F]DCFPyL PET/CT	Anti-3-[^18^F]FACBC PET/CT	$60,013.42/QALY	
[^18^F]DCFPyL PET/CT	CT C/A/P + bone scan	$66,874.17/QALY	

ICERs are listed in the table below.

## Discussion

When a newly FDA-approved diagnostic agent such as [^18^F]DCFPyL is introduced, there are many considerations that can limit its benefit in population cancer control. These include the interconnected issues of cost and race particularly when done in the context of the United States healthcare landscape. The challenges of regulatory restrictions, race, cost and access in the United States, in fact, made comparisons to extensive work performed outside of the United States quite challenging. In this study limited to our institutional catchment, we found that PSMA PET/CT imaging was disproportionately performed on EA men (age > 70 years). A higher proportion of AA patients were scanned as participants in a clinical trial than other cohorts. Irrespective of race/ethnicity, most men receiving PSMA PET/CT at our institution resided in a zip code where the median household income was greater than $100,000. This demonstrates that despite favorable insurance coverage, access was still limited for individuals who were not EA or those not living in a high-income zip code. Areas in which commercially available [^18^F]DCFPyL PET/CT was offered in the United States were restricted to institutions located in mostly wealthy neighborhoods with an EA predominance. This may explain, in part, the lack of accessibility for non-EA patients and/or those of lower socio-economic status.

It should be noted that the cost-effectiveness analysis was conducted during a very specific time interval and the landscape will change substantially as alternate radiotracers and adjusted costs are apparent from the time of publication of this manuscript. Despite this, our cost-effectiveness analysis demonstrated that there were marked variation in total costs and QALYs associated with the three imaging modalities compared: anti-3-[^18^F]FACBC PET/CT, [^18^F]DCFPyL PET/CT, and conventional imaging. [^18^F]DCFPyL PET/CT imaging can detect aggressive disease to a greater degree than the other noted imaging modalities. For example, sub centimeter lymph nodes which do not appear morphologically suspicious on conventional imaging can be PSMA avid and suggestive of metastatic disease. As a result, [^18^F]DCFPyL PET/CT was associated with higher interventional costs and higher mean composite QALY per simulation. Conventional imaging with CT chest, abdomen, and pelvis with bone scintigraphy was associated with the lowest cost and had no difference in composite lifetime QALY compared to anti-3-[^18^F]FACBC PET. However, finding aggressive disease on conventional imaging was dependent on morphological characteristics of lymph nodes and abnormal lesions on CT, as well as PSA levels greater than 10 ng/mL with bone scintigraphy in initial staging. As such, early disease may not be as obvious on first attempt, especially in the setting of low PSA levels, for individuals opting for conventional imaging for prostate cancer staging. Consequently, true overall costs would be greater for patients in this cohort.

ICER analysis demonstrated that [^18^F]DCFPyL PET/CT was more effective and more costly than the other two imaging modalities. Neither anti-3-[^18^F]FACBC PET/CT nor conventional imaging were associated with improved QALY relative to [^18^F]DCFPyL PET, as such they were not dominating in the analysis. In fact, there is scope to suggest that [^18^F]DCFPyL PET/CT may in fact still be a worthwhile option to pursue even though the ICER exceeded the willingness-to-pay threshold selected, in part due to the total costs incorporating both imaging and medical costs for the potential lifetime of the patient. This contrasts with published health economic literature where the willingness-to-pay ceiling ratio was in reference to a solitary treatment option. The costs associated with each imaging modality, or the variable of interest in this analysis, were well below the ceiling ratio (Table 1) independently. The increased costs associated with [^18^F]DCFPyL PET/CT were in fact attributed to the imaging studies ability to detect micro-metastasis at an earlier state, resulting in more aggressive therapy options and subsequently improved composite lifetime QALY. As micro-metastases are detected at an earlier state of disease, particularly at initial staging, the total costs associated with lifetime management may be decreased consequential to a reduction in likelihood of biochemical recurrence.

There is currently no published study demonstrating a comparison of all three imaging studies regarding detection at similar clinical timepoints. Therefore, we acknowledge a limitation that the extracted proportions used in this model may not accurately depict real life proportions. The estimated proportions of N1M0 and M1 disease were assumed to be the same for each modality regardless of the risk stratification in the absence of literature suggesting otherwise at initial staging. This constrains our interpretation as the outcomes of the analysis are dependent on these proportions and assumptions. Our model also does not consider the need for repeat imaging at select time intervals, as standardized time intervals for repeat imaging was not clearly advised in the NCCN guidelines and remains at clinical discretion. This was a major limitation for biochemical recurrent patients who may not have disease detected on their first set of imaging, underrepresenting overall treatment costs associated. However, to accommodate for this, the simulation follows the NCCN guidelines such that the decision to treat may be made in the absence of radiographic evidence of recurrent disease.

Another limitation of this cost-effectiveness analysis is the inability to appropriately characterize effects of interventions. Our simulation extracted HRQoL based on published literature utilizing PC-related surveys; however, these interpretations are intrinsically subjective and can be misleading. Unfortunately, we could not replace these HRQoLs with another effects-based model when calculating the ICER, as there was no standardized description in the published literature of an alternative. Serum PSA levels typically decrease after almost any PC-targeted intervention, but the extent and rate varies per person, which also makes it a poor alternative. To account for this, HRQoLs were standardized for each health transition state among all simulations run. In congruence with published literature, we demonstrated that [^18^F]DCFPyL PET/CT is a reasonable alternative to another prostate cancer approved PET/CT radiopharmaceutical.

These observations are the clinical reality in the United States as these are among the very few diagnostic PET tools at the clinician’s disposal to improve population cancer control. Under these constraints, our findings are separated from other non-United States based studies in the published literature. We found that in our catchment, [^18^F]DCFPyL PET/CT was associated with a higher QALY than anti-3-[^18^F]FACBC PET, and improved detection of aggressive disease relative to conventional imaging. As such, it would be important for more centers in the United States to implement this imaging study option without fear of extensive financial toxicity even in areas with unfavorable payer demographics. In addition, the arrival of new PSMA based radiotracers and cost competition could help improve access and mitigate healthcare disparities currently known to exist in the United States.

## Supplementary Information

Supplementary Figure 1.

## Data Availability

Publicly available data was utilized for this study. Please email the corresponding author for clarification on the data utilized. Data for the demographics study is available on request from the authors within compliance defined by institutional and federal regulations.
