# Supplementary material for: Complex implementation factors demonstrated when evaluating cost-effectiveness and monitoring racial disparities associated with [18F]DCFPyL PET/CT in prostate cancer men
Source: Sci Rep. 2023 May 23;13:8321. doi: 10.1038/s41598-023-35567-w (PMC10205741; doi:10.1038/s41598-023-35567-w)
Supplement: Supplementary file 1 — Supplementary Figure 1. [file 41598_2023_35567_MOESM1_ESM.docx]

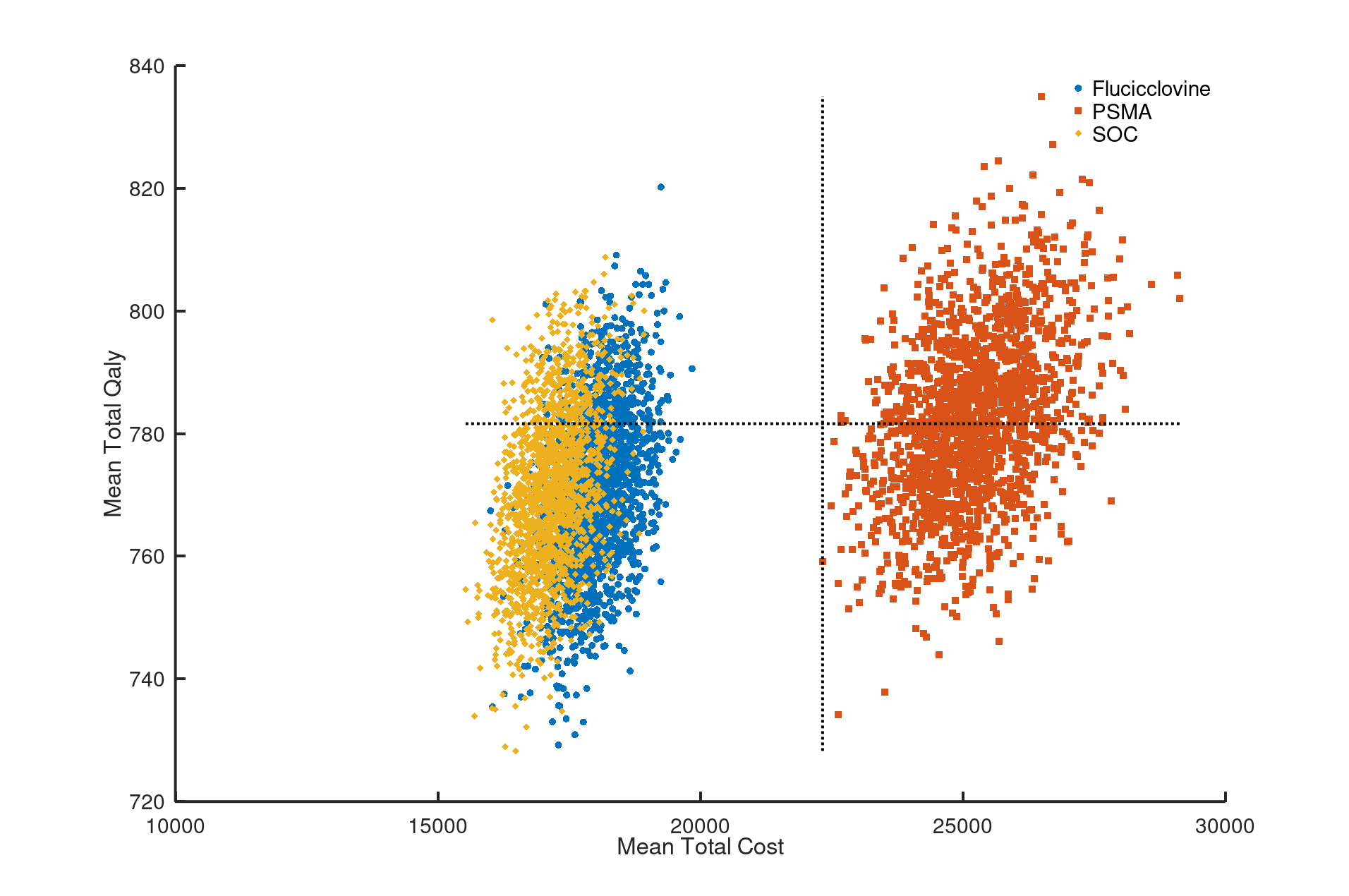


Supplemental Figure 1. Mean Total Cost and mean HRQoL for each iteration of the simulation demonstrated good precision.
